# *FMR1* premutation with Prader–Willi phenotype and fragile X‐associated tremor/ataxia syndrome

**DOI:** 10.1002/ccr3.834

**Published:** 2017-03-23

**Authors:** Verónica Martínez‐Cerdeño, Mirna Lechpammer, Stephen Noctor, Jeanelle Ariza, Paul Hagerman, Randi Hagerman

**Affiliations:** ^1^ Department of Pathology and Laboratory Medicine UC Davis Medical Center Sacramento CA USA; ^2^ Institute for Pediatric Regenerative Medicine and Shriners Hospitals for Children Northern California Sacramento CA USA; ^3^ MIND Institute UC Davis Medical Center Sacramento CA USA; ^4^ Department of Psychiatry and Behavioral Sciences UC Davis Medical Center Sacramento CA USA; ^5^ Department of Biochemistry and Molecular Medicine UC Davis Medical Center Sacramento CA USA; ^6^ Department of Pediatrics UC Davis Medical Center Sacramento CA USA

**Keywords:** *FMR1*, Fragile X, FXTAS, inclusions, neurons, Prader–Willi

## Abstract

This is a report of *FMR1* premutation with Prader–Willi phenotype (PWP) and FXTAS. Although the PWP is common in fragile X syndrome (FXS), it has never been described in someone with the premutation. The patient presented intranuclear inclusions, severe obesity, hyperphagia, and ADHD symptoms, typical of the PWP in FXS. In addition, the autopsy revealed multiple architectural cortical abnormalities.

## Graphical abstract

This is a report of the *FMR1* premutation with Prader–Willi phenotype (PWP) and FXTAS. Although the PWP is common in fragile X syndrome (FXS), it has never been described in someone with the premutation. The patient presented intranuclear inclusions, severe obesity, hyperphagia, and ADHD symptoms, typical of the PWP in FXS. In addition, the autopsy revealed multiple architectural cortical abnormalities.

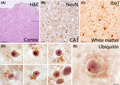

## Introduction

The Prader–Willi phenotype (PWP) of the fragile X syndrome (FXS) has been described at least in 27 patients 1, 2, 3. These patients suffered from obesity starting in childhood together with lack of satiation and hyperphagia. They also demonstrated severe behavioral problems and intellectual disability with social deficits. Patients with FXS and PWP suffered more severe behavioral problems with a higher rate of social deficits compared to FXS without the PWP 3. FXS is a neurodevelopmental syndrome with a CGG expansion of more than 200 repetitions in the *FMR1* gene, while some older carriers of this expansion (55–200 repetitions) suffer from the neurodegenerative fragile X‐associated tremor/ataxia syndrome (FXTAS). *FMR1* protein (FMRP) interacts with the mRNA translation regulators CYFIP1 and CYFIP2 4, 5. Interestingly, *CYFIP1* is localized to the region critical for Prader‐Willi Syndrome (PWS) at 15q 6. While *FMR1* messenger RNA (mRNA) and FMRP are lacking in FXS, FMRP levels are normal or mildly reduced while mRNA is increased in FXTAS.

## Case Report

FXTAS is a late‐onset neurodegenerative disorder associated with premutation alleles (55–200 CGG repeats) of the *FMR1* gene 7, 8. FXTAS is characterized by the presence of ubiquitin‐positive inclusions in neurons and astrocytes. FXTAS symptoms include cerebellar ataxia, tremor, cognitive deficits, peripheral neuropathy, autonomic dysfunction, and psychiatric involvement.

**Figure 1:**
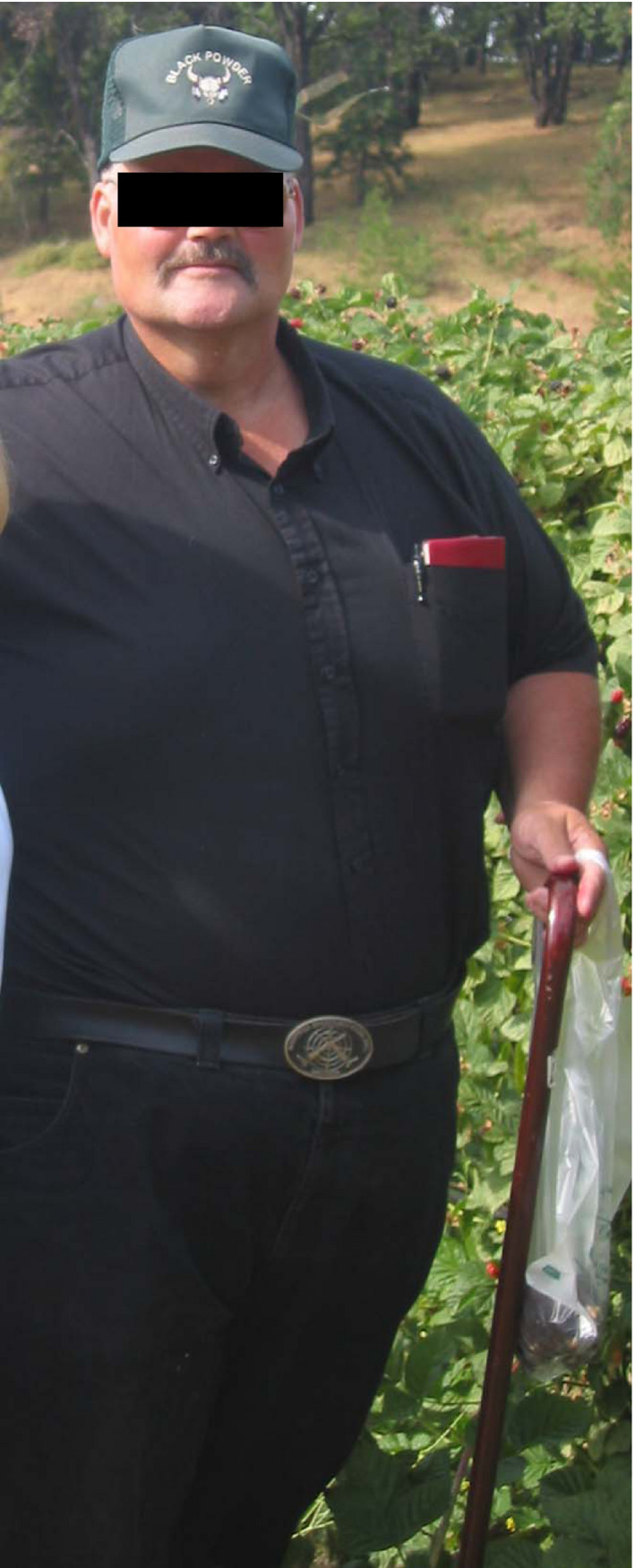
Patient's photograph.

Here, we present a male subject with features of PWP that carried an *FMR1* premutation with 73 CGG repeats and an FMRP level of 89.5% of normal. Unfortunately, samples for molecular analysis were not collected. In the future, it will be important to obtain whole‐exome sequencing of patients with FXTAS who also present with PWP. He was described as hyperactive with learning problems in school. Although he was impulsive with attention deficit disorder association (ADHD) symptoms, he never utilized stimulant medication. He had significant learning problems in school and did not graduate from high school although he received his degree years later. Anxiety was severe in childhood, and he chronically chewed on his nails. He began to gain weight at 6 years of age related to compulsive eating and lack of satiation after meals. He went to a weight control program camp at age of 8 years, but he continued to gain weight. He would eat until he threw up between 7 and 9 years old. Obesity was a chronic and significant problem for him and he underwent gastric (bypass) surgery in his forties when he weighed 400 pounds. Following surgery, he suffered indigestion and diarrhea, but he did loose over 100 lbs. He had his first myocardial infarction in his mid‐30s, suffered from multiple myocardial infarctions during his 40s, and had coronary bypass surgery in his late 40s after multiple stents had been placed. He was a smoker for many years until his early 40s. He also developed gait ataxia and began using a cane in his late 40s and 50s. He did not have a tremor but experienced handwriting problems. He was hypertensive, and experienced chronic pain in his 40s, which included migraine headaches and leg pain, the latter thought to be secondary to a neuropathy. He suffered from depression but was not treated with antidepressants. He died at age 55 in a car accident. His mother, an obligate carrier, suffered from features of FXTAS including gait ataxia leading to use of a wheelchair, neuropathic pain, and cognitive decline. His sister is a premutation carrier, and she has a son with fragile X syndrome and autism (Fig. 1).

**Figure 2:**
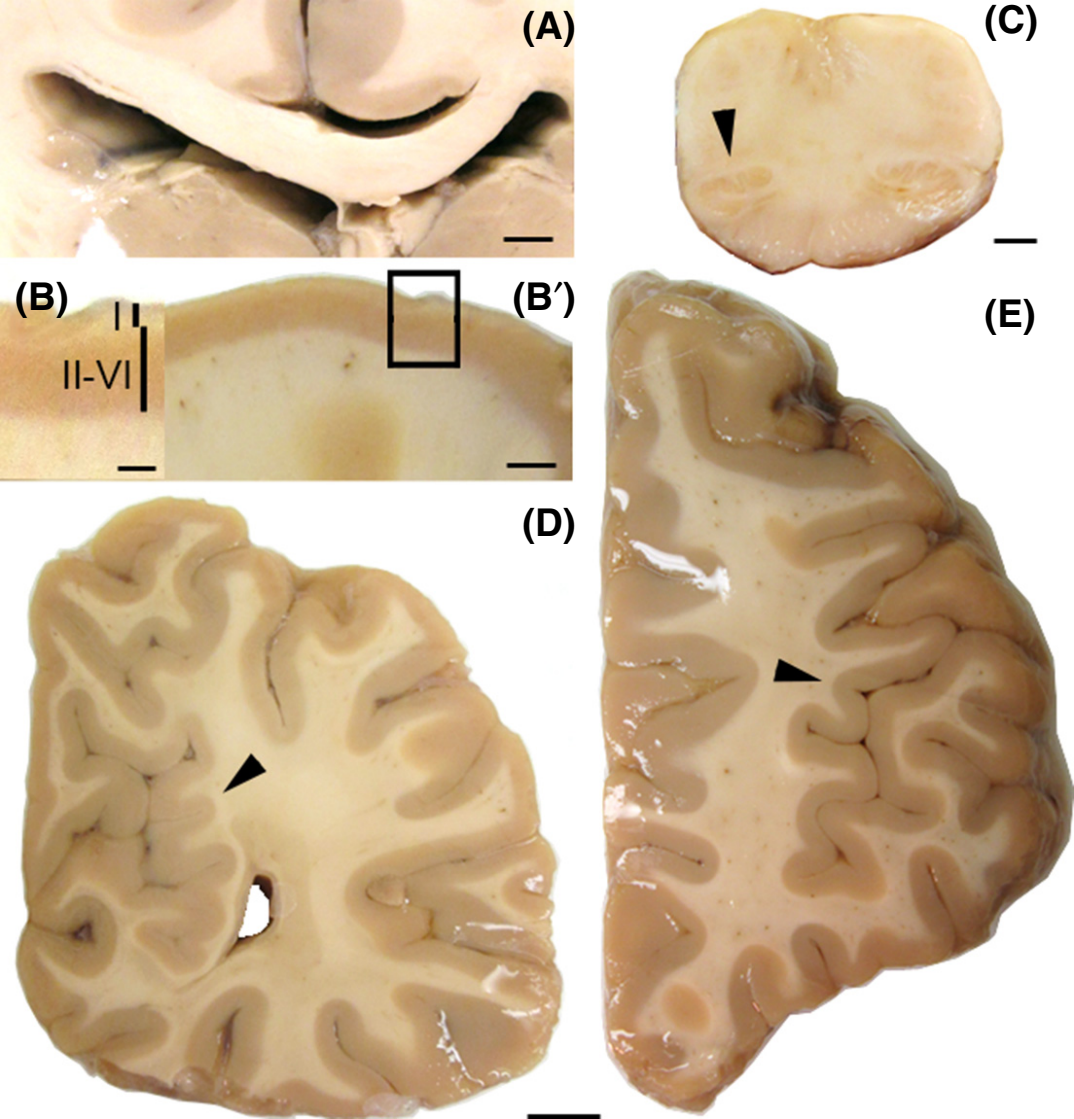
Thick coronal sections of the brain. (A) Thin corpus callosum and expanded left lateral ventricle. (B and B’) Thin cortex and thin II‐VI layers when compared to the thickened layer I. (C) Small left inferior olive. (D) Gyri crowding (arrowhead) in the medial region of the occipital lobe. (F) Abnormal gyrification (arrowhead) in the lateral frontal lobe. Calibration bars: A: 6 mm, B: 4 mm, C. 2 mm, D–E: 1 cm.

**Figure 3:**
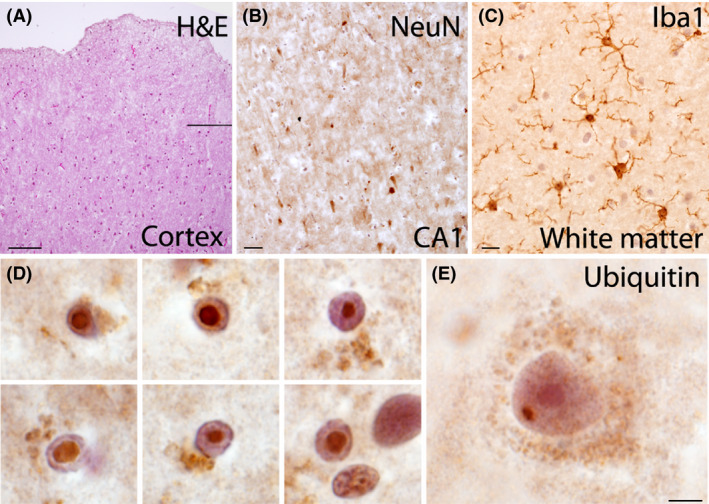
(A) H&E demonstrating abnormal undulation and thickened layer I in the temporal cortex. (B) Loss of NeuN+ pyramidal cells in the CA1 of the hippocampus. (C) Abnormally large number of Iba1 + microglial cells with an activated morphology within subcortical white matter. (D) Ubiquitin+ intranuclear inclusions within astrocyes and (E) within a pyramidal cell in the prefrontal cortex. Calibration bars: A: 200 *μ*m, B: 50 *μ*m, C. 25 *μ*m, D‐E: 10 *μ*m.

The autopsy findings were remarkable for multiple architectural cortical abnormalities including bilateral abnormal gyration or gyri crowding; mild enlargement of the left lateral ventricle; broad thinning of the cortex, more prominent in rostral cortex (3 mm); thin corpus callosum, mainly at its posterior level (0.4–0.5 mm); and a left small inferior olive (Fig. 2). The cerebral cortex presented with increased thickness, cellularity and abnormal undulation of the molecular layer, and a relative decrease in cortical thickness in layers II‐VI with marked decrease in number of cortical neurons (Fig. 3). These changes have been described previously as characteristic of PWS 9. However, other dysmorphic features of the brain described in PWS, such as cerebellar coning, were not found. The cortex presented with marked neuronal depletion, with residual neurons often exhibiting abnormal morphology. Prominent gliosis was observed and highlighted with GFAP and Olig2. Numerous Iba1‐positive microglia were observed. There were no beta‐amyloid plaques or tau‐associated pathology and no iron accumulation. White matter was hypercellular and populated by microglia (highlighted by Iba‐1) and reactive astrocytes (GFAP+). In addition, intraparenchymal blood had perivascular clearing and hyalinized walls. The ependyma appeared focally denudated with underlying subependymal gliosis. There was a loss of pyramidal neurons in the CA1 with associated gliosis. Some neurons exhibited abnormal perikaryon accumulation of SMI312 that also highlighted abnormally thickened axons. The cerebellar cortex possessed a thickened molecular layer, and cerebellar Purkinje cells manifested moderate patchy depletion. The dentate nucleus had prominent acutely ischemic red neurons. There were also findings consistent with hypoxic/ischemic injury of the brain such as small vessel disease that is usually seen in patients with hypertension or diabetes.

Ubiquitin‐positive intranuclear inclusions were present in astrocytes and neurons, consistent with a diagnosis of FXTAS. Cerebral and cerebellar cortices presented with intranuclear inclusions in 14% of cells, while the hippocampus had 3% in the molecular layer, 14% in the pyramidal layer, and 11% in the granular layer. Intranuclear inclusions in astrocytes were massive, occupying up to 80% of the nucleus, while in neurons, the inclusions were smaller and about the size of the nucleolus (Fig. 3D and E).

## Discussion

This is the first case linking the presence of the *FMR1* premutation to PWP. This case presented with severe obesity, severe hyperphagia, lack of satiation, and ADHD symptoms, typical of the PWP described in FXS 3. The premutation involvement manifested with FXTAS symptoms that began in his 40s, unusually early for the symptoms of neuropathy and ataxia. He meets criteria of FXTAS based on the presence of the intranuclear inclusions throughout his brain, in addition to symptoms of ataxia and neuropathy 10. In addition, the autopsy findings included multiple architectural cortical abnormalities that may be related to migration problems 11. These problems are likely additive to his history of ADHD and learning problems in childhood. Such learning/developmental (ADHD, social deficits, and anxiety/depression) problems occur in approximately 15–30% of premutation males 12, 13. He also experienced early onset of FXTAS symptoms, which may be more likely to occur if developmental problems are experienced in childhood such as ASD. At 55 years, the number and size of the inclusions were very high compared to that of other premutation carriers previously described 14, 15, 16. It is likely that his recurrent hypoxia from repeated myocardial infarctions and his hypertension increased white matter disease and that exacerbated the progression of FXTAS. It may also be possible that the presence of the PWP and depression added increased oxidative stress that may also have exacerbated the progression of FXTAS 17, 18. Other cases of the PWP have only been reported with patients with FXS 2, 3. However, his mildly low level of FMRP could have led to predisposition toward the PWP. FMRP controls *CYFIP* which is located in the 15q deletion region of PWS and a previous report demonstrated downregulation of *CYFIP 1* in the PWP in FXS 3. Clinically he did not meet criteria for PWS because his learning problems were not severe or typical for PWS and he had frequent vomiting in childhood which does not happen in those with PWS. There may be more cases of the PWP in premutation carriers particularly those with low levels of FMRP combined with developmental and eating problems at an early age as reported here 5.

The interaction between an *FMR1* mutation and the PWP is worthy of further exploration.

## Conflict of Interest

The funding source had no role in the design and conduct of the study; collection, management, analysis, or interpretation of the data; preparation, review, or approval of the manuscript; and decision to submit the manuscript for publication.

## Authorship

VMC: designed the study, acquired data, analyzed and interpreted data, made figures, wrote the manuscript, and supervised study. ML: analyzed and interpreted data. JA: acquired data. SN and PH: reviewed the manuscript for intellectual content. RH: designed the study, acquired data, analyzed and interpreted data, reviewed the manuscript for intellectual content, and supervised the study.
